# Reactive infectious mucocutaneous eruption secondary to Chlamydia pneumoniae infection in a 19-year-old: a case report

**DOI:** 10.1099/acmi.0.001160.v3

**Published:** 2026-05-05

**Authors:** Cole Schonhofer, Divya Santhanam, Jollee S.T. Fung, Natasha Press

**Affiliations:** 1 Department of Pathology and Laboratory Medicine, University of British Columbia, Vancouver, British Columbia, Canada; 2 Department of Medicine, University of Toronto, Toronto, Ontario, Canada; 3 Division of Infectious Diseases, Department of Medicine, University of British Columbia, Vancouver, British Columbia, Canada

**Keywords:** *Chlamydia pneumoniae*, pneumonia, reactive infectious mucocutaneous eruption

## Abstract

**Introduction.** Reactive infectious mucocutaneous eruption (RIME) is a phenomenon that occurs predominantly in children and young adults following a bacterial or viral respiratory infection. RIME is most associated with *Mycoplasma pneumoniae* and generally presents with extensive mucosal and limited cutaneous involvement. Less commonly, infection with *Chlamydia pneumoniae* can trigger cutaneous eruptions but seldom leads to isolated mucosal involvement. Here, we describe a rare case of RIME mucositis secondary to *C. pneumoniae* respiratory infection.

**Case report.** An otherwise healthy 19-year-old male presented with fever, cough, conjunctivitis, dysuria and oral mucositis with ulceration preventing food intake. *C. pneumoniae* was detected on a nasopharyngeal swab by a commercial multiplex PCR (BioFire Respiratory 2.1 Panel), while a broad workup of alternative infectious and autoimmune causes was unremarkable. Despite supportive care, antibiotics and initial steroid treatment, his mucositis necessitated hospital admission and eventually steroid regimen intensification before fully resolving.

**Conclusion.** While rare, *C. pneumoniae* should be considered as a possible trigger of RIME in adolescents and young adults. Multiplex PCR assays capable of detecting atypical pneumonia pathogens such as *C. pneumoniae* and *M. pneumoniae* can assist in diagnosing patients presenting with RIME-compatible symptoms. Treatment involves supportive care, antibiotics and steroids if mucositis is severe. Steroid escalation may be beneficial in patients with slow-resolving mucositis.

## Data Summary

No data were collected for this case report.

## Introduction

Reactive infectious mucocutaneous eruption (RIME) occurs predominantly in children and adolescents [1,3]. Most cases present with oral mucositis, followed by ocular and genitourinary in order of frequency [4]. It can be differentiated from Stevens–Johnson syndrome (SJS) and toxic epidermal necrolysis (TEN) as it classically follows respiratory infection rather than drug exposure, has limited cutaneous involvement, and has different underlying pathogenesis and clinical outcomes. While RIME has classically been linked to *Mycoplasma pneumoniae* infection, other respiratory pathogens have also rarely been implicated. In particular, *Chlamydia pneumoniae* can also trigger mucocutaneous eruptions; however, unlike with *M. pneumoniae*, lesions are predominantly cutaneous [5,8]. While the prognosis of RIME is generally excellent, mucositis complications, such as dysphagia, can require hospitalization, supportive therapy and steroid treatment. As such, recognition and diagnosis are important to guide clinical decision-making on further treatment options. Here, we present a rare case of RIME with severe mucositis triggered by *C. pneumoniae* infection.

## Case presentation

A 19-year-old male presented to the hospital after 4 days of fever, productive cough, sore throat and conjunctivitis. One day after symptoms started and 3 days prior to hospital presentation, his family physician had prescribed clarithromycin 500 mg BID and doxycycline 100 mg BID for presumed community-acquired pneumonia (CAP). His symptoms persisted despite antibiotics, and he developed new oral ulcerations associated with odynophagia and anorexia, along with new dysuria.

He had no significant past medical history or allergies and was fully vaccinated. Other than the recently prescribed antibiotics, he was not taking any additional medications. He had no recent travel or animal exposure other than a pet dog. He did not have any sexual or substance use history and was employed as a mechanic.

**Fig. 1.**
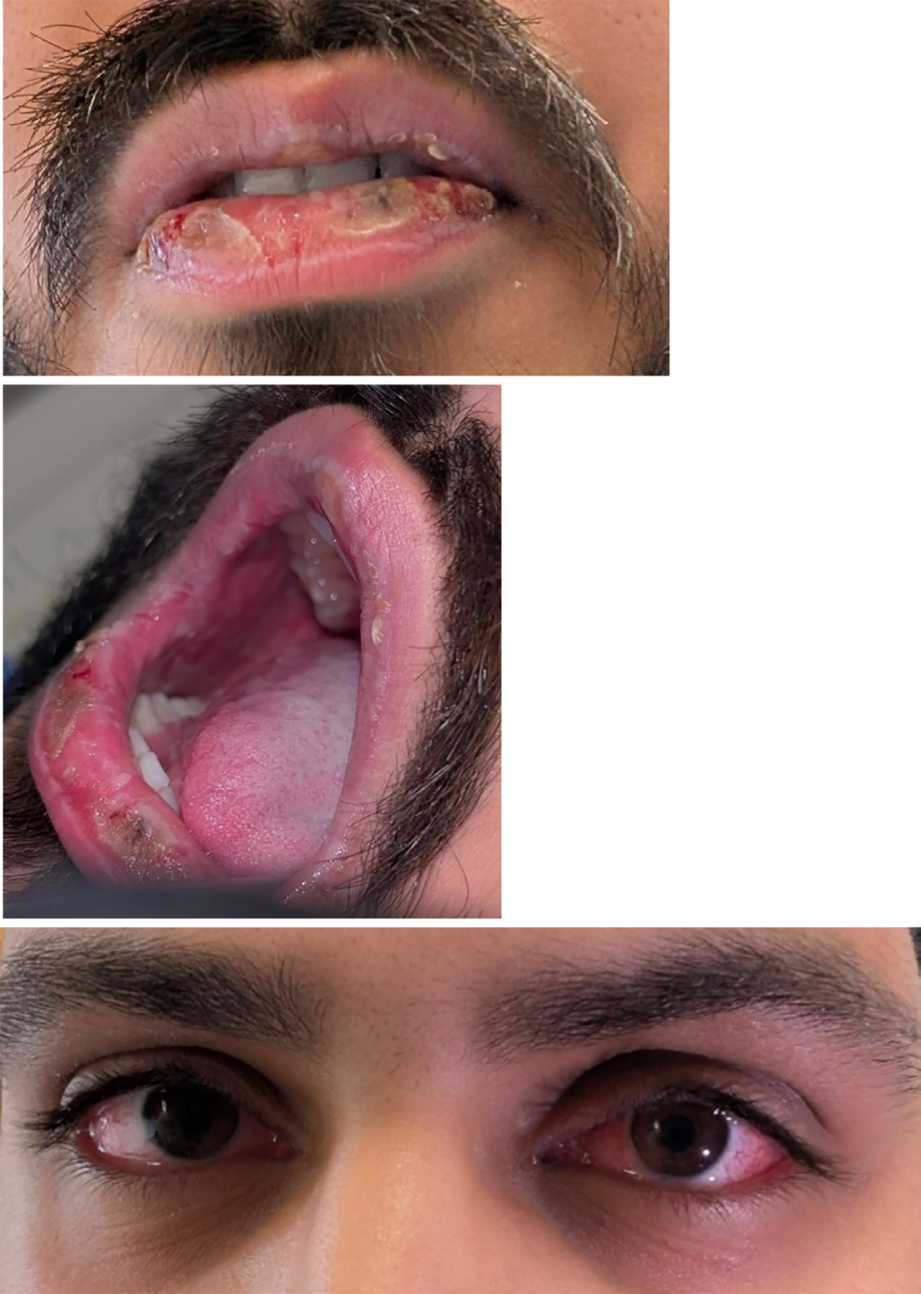
Photos of non-vesicular lip and oral ulceration and non-purulent conjunctivitis, taken 1 day after presentation.

On assessment, he was febrile with a temperature of 38.2 °C and tachycardic with a heart rate of 119. He was normotensive and was not in respiratory distress. Non-vesicular ulcerations were noted on his lips and oral cavity, and conjunctival erythema was present bilaterally without discharge or exudate (Fig. 1). He had mild cervical lymphadenopathy. Cardiac exam revealed no murmurs, rubs or peripheral oedema. On lung auscultation, there were decreased breath sounds and crackles to the right side. His abdomen was soft and non-tender. There was erythema to the penile meatus but no discharge or ulceration. There were no cutaneous lesions or rash.

**Fig. 2.**
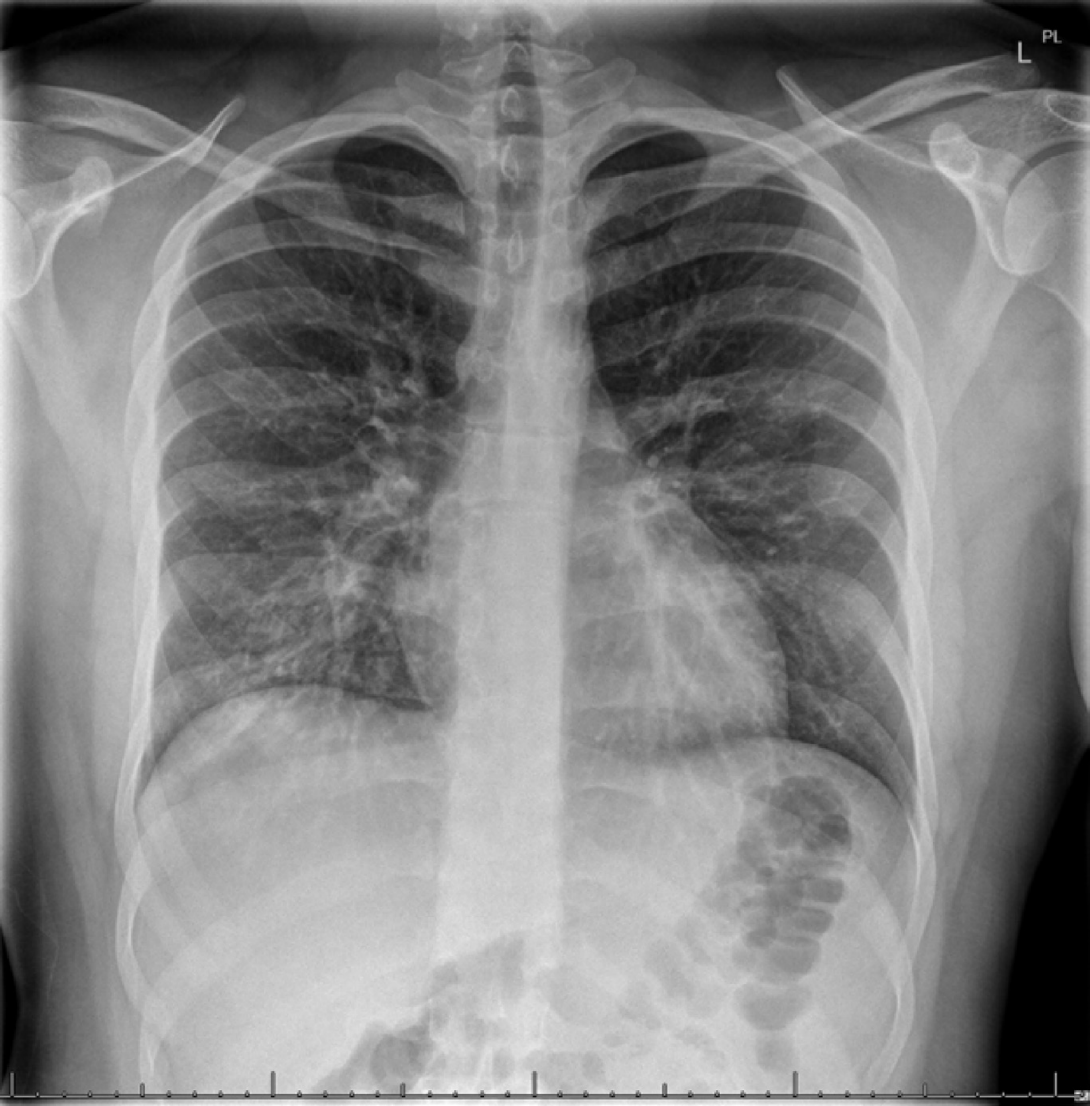
Chest X-ray taken on admission, demonstrating right lower lobe consolidation consistent with CAP.

Initial investigations demonstrated mild leukocytosis at 13.6×10^9^ l^−1^ (reference range 4.0–11.0×10^9^ l^−1^), hypokalaemia of 2.8 mmol l^−1^ (3.5–5.0 nmol l^−1^), elevated creatinine of 118 µmol l^−1^ (50–95 µmol l^−1^) and elevated C-reactive protein of 126 mg l^−1^ (<2.1 mg l^−1^). Urinalysis was negative for nitrites and leucocyte esterase, while chest X-ray demonstrated focal consolidation through the right lower lung zone (Fig. 2). He was diagnosed with CAP and acute kidney injury, started on ceftriaxone 2 g IV daily and oral azithromycin 500 mg daily, given IV fluids, and admitted to the hospital. The differential was broad and included bacterial pneumonia, viral infections including measles, adenovirus, respiratory syncytial virus, and severe acute respiratory syndrome coronavirus 2 (SARS-CoV-2), acute HIV mucositis, and gonococcal/chlamydial urethritis. Non-infectious causes of mucositis, including SJS and TEN in the context of recent antibiotic use, were also considered. Ultimately, his presentation was felt to be in keeping with RIME following a respiratory infection. SJS/TEN was considered unlikely as he had started developing mucositis and conjunctivitis prior to starting antibiotics, he had no cutaneous lesions or skin detachment, typically seen in SJS/TEN and his severity-of-illness score for toxic epidermal necrolysis was 0.

Initial investigations included blood, sputum, conjunctival, and throat bacterial cultures, oral swabs for herpes simplex 1/2 and varicella zoster virus PCR, conjunctival swab for adenovirus PCR, HIV antibody/antigen enzyme immunoassay and viral load PCR and chlamydia/gonorrhoea PCR from urine. Measles PCR from urine and nasopharyngeal swab were also requested. Additionally, a nasopharyngeal BioFire multiplex respiratory PCR (bioMérieux, Marcy-l'Étoile, France) was ordered. All investigations returned negative other than the BioFire respiratory panel, which was positive for *C. pneumoniae*. His sputum also subsequently tested positive for *C. pneumoniae* via the BioFire panel, while a throat swab was negative. Other panel targets, including *M. pneumoniae*, were negative, and the results were confirmed at the provincial reference lab via a separate, lab-developed PCR assay. Serology for *C. pneumoniae* and *M. pneumoniae* was not performed.

The patient was started on dexamethasone 3 mg IV daily for mucositis treatment (equivalent to 0.2 mg/kg/day prednisolone) and continued ceftriaxone and azithromycin for pneumonia. Respiratory symptoms improved after 5 days, and antibiotics were stopped. However, odynophagia and dysuria continued despite steroid and topical oral lidocaine treatment, and he was unable to tolerate solid food intake. He also developed new scrotal and glans penis ulcers. Dermatology was consulted, and they agreed with the RIME diagnosis. His steroids were intensified to 45 mg IV methylprednisolone (equivalent to 0.6 mg/kg/day prednisolone) with 5 mg/ml prednisolone oral rinses and clobetasol ointment for his lips. After 4 days of higher-dose and topical steroid treatment, his odynophagia, dysuria and genital ulcers improved, and he was discharged home on a 15-day oral prednisone taper with dermatology follow-up.

## Discussion

*C. pneumoniae* is an obligate intracellular respiratory pathogen transmitted by droplets, with a prolonged 3-4 week incubation period compared to typical respiratory viruses [9]. Serologic epidemiological studies have found that up to 70–80% of people are exposed to *C. pneumoniae* by the age of 70, and it is implicated in up to 10% of CAP cases, although coinfection with other respiratory pathogens is common [9,10]. Most infections are asymptomatic or mild with persistent cough, low-grade fever and malaise, although severe disease can occur in elderly or immunocompromised patients and in those with underlying respiratory conditions, such as asthma and COPD. Additionally, *C. pneumoniae* has been implicated in the development of chronic diseases, including atherosclerosis [9].

Traditionally, serology has been the gold standard of *C. pneumoniae* diagnosis. More recently, PCR-based methods have enabled rapid and accurate diagnosis [9]. In Canada, the Biofire respiratory 2.1 panel is approved for use on respiratory samples and includes *C. pneumoniae* as well as other bacterial and viral respiratory pathogens, including *M. pneumoniae*, as targets. In our patient’s case, this multiplex assay allowed rapid identification of *C. pneumoniae* and confirmation of RIME diagnosis.

While there are no specific guidelines for *C. pneumoniae* treatment, it is one of the atypical pathogens covered by CAP regimens that include macrolides such as azithromycin, fluoroquinolones, or doxycycline [11]. It is intrinsically resistant to sulphonamide antibiotics, including trimethoprim-sulfamethoxazole. Beta-lactam antibiotics have traditionally been considered inferior for intracellular pathogens such as *C. pneumoniae*, although a recent meta-analysis found no difference in outcomes in non-severe CAP [12]. A 5-day course of azithromycin, as used in our case, has demonstrated clinical and microbiological efficacy [9].

A 2017 literature review by Mayor-Ibarguren *et al*. found 20 reported cases of *C. pneumoniae* infection with mucocutaneous manifestations [8]. Interestingly, they found that most cases presented with cutaneous manifestations such as maculopapular rash or targetoid lesions, while only seven cases featured mucosal lesions and only two had mucositis without cutaneous involvement. In comparison, mucocutaneous eruptions following *M. pneumoniae* infection were much more commonly limited to the mucosa. Similarly, recent literature reviews by Di Luigi *et al*. found 47 published reports of *C. pneumoniae*-related cutaneous eruptions but only 5 reports featuring exclusive mucosal lesions [6,7]. In contrast, they found 53 reports of exclusive mucosal involvement related to *M. pneumoniae* infection.

Mucocutaneous eruptions related to *M. pneumoniae* have historically been referred to by various terms, including SJS, atypical SJS, TEN, Fuchs syndrome, erythema multiforme and *M. pneumoniae*-associated mucositis. In 2015, Canavan *et al*. proposed the term mycoplasma-induced rash and mucositis (MIRM) and introduced diagnostic criteria including involvement of two or more mucosal sites and <10% body surface area, with clinical and microbiological evidence of atypical pneumonia [4]. Various other bacterial and viral pathogens have also rarely been implicated in mucocutaneous eruptions, including *C. pneumoniae*, SARS-CoV-2, influenza A and B and rhino/enterovirus, among others [1,3, 5, 6, 8]. As such, the term RIME was introduced to encompass MIRM as well as other infectious triggers [3]. Diagnostic criteria are summarized in Table 1.

**Table 1.** Diagnostic and treatment criteria for RIME

Diagnosis [1,4]	Requires all of the following: Erosive mucositis in ≥2 sites (especially oral, ocular, genitourinary in order of likelihood) Recent or concurrent respiratory infection Absence of medication exposure or lesion morphology concerning for SJS/TEN Supporting RIME diagnosis: Detection of pathogens, including *M. pneumoniae, C. pneumoniae,* or respiratory viruses Cutaneous involvement is absent or polymorphic and limited to <10% body surface area
Treatment [1,8]	Supportive care, including skin and mucosal care, pain control, fluids, and nutrition management Macrolide, doxycycline, or fluoroquinolone antibiotics if there is a clinical diagnosis of bacterial pneumonia and/or *M. pneumoniae* or *C. pneumoniae* are detected Consider systemic and topical steroid treatment for moderate/severe mucositis Consider infectious diseases and/or dermatology consult for diagnosis and treatment guidance Consider ophthalmology consult for moderate/severe conjunctivitis with visual changes

In comparison to RIME, SJS/TEN also features mucocutaneous eruptions but typically has a worse prognosis and a different pathogenesis. SJS/TEN is a type IV delayed-type hypersensitivity reaction almost always triggered by medication [13]. Classically associated antibiotics include trimethoprim-sulfamethoxazole and beta-lactams, but other antibiotics have also been rarely implicated [14]. Less commonly, viral and *M. pneumoniae* infections have also been described as triggers in the absence of offending medications [13]. Conversely, RIME pathogenesis is not thought to be mediated by type IV hypersensitivity. Rather, it has been proposed that polyclonal B cell proliferation and antibody production in response to infection lead to damage via immune complex deposition and complement activation, as in a type III hypersensitivity reaction [1,15]. Alternatively, or perhaps concurrently, molecular mimicry such as that seen between *M. pneumoniae* P1-adhesion molecules and keratinocyte antigens may also contribute to disease [15]. Unfortunately, there are no diagnostic tests to distinguish between RIME and SJS/TEN, and skin/mucosal biopsies demonstrate similar histopathologic findings [1]. Typically, RIME can be clinically distinguished from SJS/TEN based on younger patient age, preceding respiratory infection, minimal cutaneous involvement without skin detachment, and milder illness course. Ultimately, SJS/TEN is a life-threatening reaction that must be considered when diagnosing RIME, especially in patients who have received antibiotics or other higher-risk medications such as antiepileptics.

There are no established guidelines for the treatment of RIME, but recommendations are summarized in Table 1. Antibiotic treatment of the underlying respiratory infection is recommended if *M. pneumoniae* or *C. pneumoniae* is identified or if there is otherwise clinical concern for bacterial pneumonia. However, there is limited evidence that the RIME phenomenon resolves with antibiotic therapy [1,6]. Treatment for RIME itself is generally supportive, and most patients recover completely within days to weeks with or without immunosuppressive treatment. In cases where mucositis symptoms are severe enough to impact oral intake or otherwise cause significant discomfort, systemic corticosteroids should be considered [1]. While there is limited data to support dosing recommendations, reviews by Mayor-Ibarguren *et al.* and M.L. Ramien proposed short, high-dose steroid courses, such as 0.5–1 mg/kg/day prednisolone for 7 days [1,8]. Our patient initially received lower doses of dexamethasone (3 mg, equivalent to 0.2 mg/kg/day prednisolone) with limited response, before eventually responding well to intensified 45 mg/day methylprednisolone (equivalent to 0.6 mg/kg/day prednisolone), anecdotally supporting these recommendations. There are also reports of successful treatment with steroid-sparing agents, such as cyclosporine, IVIG, and anti-tumour necrosis factor alpha medications in the literature [1]. In cases with significant ocular involvement, ophthalmology assessment should also be considered.

This case demonstrates a rare presentation of RIME with severe mucositis secondary to PCR-confirmed *C. pneumoniae* infection, requiring steroid escalation for symptom resolution. While *M. pneumoniae* is most associated with RIME, clinicians should consider other pathogens, such as *C. pneumoniae*, in patients presenting with mucositis and recent respiratory infection. Multiplex molecular assays such as the BioFire respiratory 2.1 panel can rapidly and accurately detect these respiratory pathogens with a single test and provide microbiologic evidence for RIME diagnosis. Further, our case provides anecdotal support for steroid treatment at doses of 0.5–1 mg/kg/day prednisolone or equivalent for moderate/severe mucositis, with escalation if needed.

## Abbreviations

CAP: community-acquired pneumonia
HIV: Humman Immunodefinciency virus
IV: intravenous
MIRM: mycoplasma-induced rash and mucositis
RIME: reactive infectious mucocutaneous eruption
SARS-COV-2: severe acute respiratory syndrome coronavirus 2
SJS: Stevens–Johnson syndrome
TEN: toxic epidermal necrolysis
